# Diabetic Retinopathy Risk in SGLT2i vs GLP1RA Initiators With CKD Stages 3-4 and Type 2 Diabetes

**DOI:** 10.1016/j.xkme.2026.101518

**Published:** 2026-09-09

**Authors:** Panupong Hansrivijit, Elisabetta Patorno, Helen Tesfaye, Deborah J. Wexler, Miin Roh, Julie M. Paik

**Affiliations:** 1 Division of Pharmacoepidemiology and Pharmacoeconomics, Department of Medicine, Brigham and Women’s Hospital and Harvard Medical School, Boston, MA; 2 Division of Renal Medicine, Department of Medicine, Brigham and Women’s Hospital and Harvard Medical School, Boston, MA; 3 Diabetes Center, Massachusetts General Hospital and Harvard Medical School, Boston, MA; 4 New England Geriatric Research, Education and Clinical Center, VA Boston Healthcare System, Boston, MA; 5 Atrius Health, Boston, MA

**Keywords:** Diabetic retinopathy, chronic kidney disease, diabetes, SGLT2 inhibitors, GLP-1 receptor agonists

## Abstract

**Rationale & Objective:**

Sodium-glucose cotransporter-2 inhibitors (SGLT2i) and glucagon-like peptide-1 receptor agonists (GLP1RA) have demonstrated renal and cardiovascular benefits among patients with chronic kidney disease (CKD) and type 2 diabetes (T2D). However, their comparative effects on diabetic retinopathy (DR) outcomes in patients with CKD stages 3-4 and T2D remain unclear. This study aimed to demonstrate the effect of SGLT2i vs GLP1RA in this population.

**Study Design:**

Population-based, new-user, active comparator cohort study comparing SGLT2i versus GLP1RA initiators. Subjects were divided into 2 cohorts: (1) incident nonproliferative diabetic retinopathy (NPDR) and (2) DR progression cohort.

**Setting & Study Population:**

Patients with CKD stages 3-4 and T2D across 3 US health care databases: Optum’s de-identified Clinformatics Data Mart Database (2014-2024), Merative MarketScan (2014-2022), and Medicare Fee-for-Service (2014-2020).

**Selection Criteria for Studies:**

Adults with a new prescription for either SGLT2i or GLP1RA without prior use in the preceding 365 days. The primary outcomes were incident NPDR and DR progression, identified through ICD-9/10 diagnosis codes and procedural codes for retinopathy treatments.

**Data Extraction:**

Exposures, covariates, and outcomes were obtained from structured claims fields.

**Analytical Approach:**

Both cohorts were analyzed using propensity score matching (1:1) to control for confounding. Incidence rates (IRs), rate differences, and hazard ratios (HRs) were calculated.

**Results:**

After 1:1 propensity score matching, the study included 20,135 matched patient pairs in the incident NPDR cohort and 7,763 matched pairs in the DR progression cohort. For incident NPDR, there was no difference between SGLT2i and GLP1RA groups (IR 29.55 vs 30.38 per 1,000 person-years, respectively; HR 0.98 [95% CI, 0.87-1.10]). Similarly, DR progression was not different between groups (IR 32.73 vs 35.24 per 1,000 person-years; HR 0.92 [95% CI, 0.76-1.12]). Subgroup analyses showed no differences in HRs for both incident NPDR and DR progression across multiple predefined subgroups.

**Limitations:**

Potential for residual confounding, outcome misclassification, and limited generalizability to non-U.S. populations.

**Conclusions:**

Initiation of SGLT2i was not associated with a different risk of incident NPDR or DR progression compared with GLP1RA among patients with CKD stages 3-4 and T2D. Additional studies with extended follow-up are needed to better characterize long-term retinal safety profiles of these therapies.

## Plain-Language Summary

People with chronic kidney disease and type 2 diabetes often need medicines that protect the kidneys and heart, but it is unclear whether one of the two used drug classes would impact eye disease caused by diabetes. We studied adults starting either sodium-glucose cotransporter-2 inhibitors or glucagon-like peptide-1 receptor agonists in three US health databases. We separately examined new cases of early diabetic eye disease and worsening of existing eye disease. After matching patients with similar characteristics, we found that the 2 treatment groups had similar eye risks for both outcomes during the follow-up period. These findings matter because they suggest eye risk may not be the main reason to choose between these therapies, although longer-term studies are still needed.

Diabetic retinopathy (DR) is a common complication in patients with diabetes, affecting approximately 26% of individuals in the United States^1^ and is expected to affect approximately 16 million Americans living with diabetes by 2050.^2^ Diabetic retinopathy and chronic kidney disease (CKD) are prominent microvascular complications of diabetes.^3^ These complications are believed to exhibit distinct structural and functional characteristics within the renal and retinal vasculature.^4^ In contrast to type 1 diabetes, where more than 90% of patients with CKD develop DR, these conditions frequently occur independently in individuals with type 2 diabetes (T2D).^5^, ^6^, ^7^, ^8^

Multiple randomized controlled trials have demonstrated the efficacy of sodium-glucose cotransporter-2 inhibitors (SGLT2is) in reducing adverse cardiorenal outcomes in patients with CKD.^9^, ^10^, ^11^ It has been suggested that SGLT2is could lower the risk of DR by inhibiting retinal sodium-glucose cotransporter 2 protein receptors.^12^ Although a post hoc analysis from the EMPA-REG OUTCOME trial suggested a numerically lower risk of composite diabetic retinopathy outcomes with empagliflozin versus placebo, the difference was not statistically significant.^13^ Similarly, a previous study of patients with T2D conducted in several claims databases, regardless of CKD status, did not find any associations between initiating empagliflozin and incident nonproliferative DR.^14^ Altogether, the risk of DR in patients with CKD and T2D remains unknown.

Glucagon-like peptide-1 receptor agonists (GLP1RAs) have also emerged as effective treatments for CKD and T2D, particularly among patients with concurrent cardiorenal conditions. The 2024 FLOW trial revealed that semaglutide significantly reduced clinically important renal outcomes and cardiovascular mortality in patients with CKD and T2D,^15^ findings consistent with the earlier SUSTAIN-6 trial that demonstrated cardiovascular benefits. However, the SUSTAIN-6 trial also noted a concerning higher incidence of retinopathy complications with semaglutide compared to placebo.^16^ In contrast, a large cohort study from the United Kingdom did not find an increased DR risk associated with GLP1RA use relative to treatment with two or more oral antidiabetic medications.^17^

Despite strong evidence supporting SGLT2is and GLP1RAs for managing CKD and T2D, uncertainty persists regarding their comparative effects on DR risk. To address this gap, we conducted a comparative effectiveness study evaluating the association between SGLT2i and GLP1RA therapies and the risk of diabetic retinopathy among patients with CKD and T2D.

## Methods

### Study Design and Data Sources

This study adopted a population-based, new-user, active comparator cohort design. Data were from 3 large US health care databases: Optum’s de-identified Clinformatics Data Mart Database (Optum CDM) (January 1, 2014 to February 29, 2024), Merative MarketScan (January 1, 2014 to December 31, 2022), and Medicare Fee-for-Service Parts A, B, and D (January 1, 2014 to December 31, 2020). Optum CDM and Merative MarketScan databases include administrative health claims from large commercial and Medicare Advantage health plan members. Medicare fee-for-service encompasses federally sponsored healthcare data of adults aged ≥65 years and individuals with disabilities. All 3 databases contain longitudinal patient-level data, such as demographics, diagnoses, medical procedures, hospital admissions, pharmacy dispensation records, and insurance enrollment details. The institutional review board at Mass General Brigham approved the study, with respective data-use agreements established for each database. Informed consent was waived due to the exclusive use of de-identified secondary data. The study reporting aligns with the Strengthening the Reporting of Observational Studies in Epidemiology (STROBE) guidelines.

### Study Population

The study included adults diagnosed with CKD stages 3-4 and T2D who were aged ≥18 years for the commercial databases (Optum CDM and MarketScan) and ≥65 years for the Medicare database. For each database, patients were included in the study if they initiated an SGLT2i or GLP1RA (without prior use in the last 365 days) during the study period. Chronic kidney disease^18^ and T2D^19^ diagnoses were identified using validated International Classification of Diseases (ICD) 9/10 codes.

We selected GLP1RA initiators as the active comparator in a new-user design to reduce confounding by indication. SGLT2i and GLP1RA are both used in overlapping CKD and T2D populations and share cardiorenal indications, making them more clinically comparable than other glucose-lowering medications, which are typically prescribed for different reasons and at different disease stages.

We generated 2 distinct retinopathy outcome-specific cohorts, Incident non-proliferative DR (NPDR) cohort and DR progression cohort. For incident NPDR cohort, eligibility required at least 1 routine vision or retinal examination, or an ophthalmology/optometry visit in the year preceding and including cohort entry to identify pre-existing DR. Individuals with prior retinopathy and treatment for retinopathy were excluded. These include history of DR, vitreous hemorrhage, vitrectomy, retinal vein occlusion, macular degeneration, prior repair or treatment of retinopathy, photocoagulation, or intravitreal anti-vascular endothelial growth factor injection (Table S1). For DR progression cohort, participants had to have a documented history of NPDR in addition to the other eligibility criteria (Table S1). Patients were excluded if any of the following are present: history of diabetic retinopathy, proliferative diabetic retinopathy, vitreous hemorrhage, vitrectomy, retinal vein occlusion, macular degeneration, prior repair or treatment of retinopathy, photocoagulation, or intravitreal anti- vascular endothelial growth factor injection.

In both cohorts, individuals were excluded if they had fewer than 365 days of continuous insurance coverage before treatment initiation, missing demographic information (age or sex), concurrent prescriptions for both study drugs, used combination drugs (metformin or other non-study agents), received tirzepatide (Mounjaro or Zepbound), semaglutide 2.4 mg (Wegovy), or liraglutide 3.0 mg (Saxenda)—formulations specifically approved for weight management—, as well a diagnosis of type 1 diabetes, secondary diabetes, history of pancreatitis, cirrhosis, multiple endocrine neoplasia type 2, bariatric surgery, organ transplantation, end-stage kidney disease, or resided in nursing homes.

### Exposure and Cohort Follow-Up

Exposure to the study medications was determined from outpatient pharmacy dispensing data. Follow-up commenced the day after cohort entry date, continuing until discontinuation of the initial drug, switching to the comparator, disenrollment from health care or pharmacy plans, or death, whichever occurred first. We selected GLP1RA as the active comparator because, similar to SGLT2i, GLP1RAs was approved by the United States Food and Drug Administration to reduce the risk of CKD progression, end-stage kidney disease, and cardiovascular death in adults with T2D and CKD.^20^ This clinical rationale was further supported by substantial overlap in the propensity score distributions between SGLT2i and GLP1RA initiators, indicating that the 2 treatment groups were highly comparable. Conversely, other potential comparators—such as DPP-4 inhibitors, insulin, and sulfonylureas—are not indicated for renal and cardiovascular protection similar to SGLT2is and GLP1RAs. There was also insufficient overlap in propensity score distributions, which could introduce significant confounding by indication if selected as an active comparator.

### Outcomes

Incident NPDR was identified by inpatient or outpatient diagnoses of mild, moderate, or severe NPDR. DR Progression was defined as initiation of retinopathy treatments (intravitreal anti-vascular endothelial growth factor injections or pan-retinal photocoagulation) or onset of vitreous hemorrhage or proliferative DR (Table S2). These outcome definitions have been used previously in peer-reviewed publications.^14^^,^^21^

### Baseline Covariates

Baseline covariates were assessed during the 365 days preceding the cohort entry date for patients in both the Incident NPDR and DR progression cohorts. These covariates included demographics (eg, age and sex), geographic locations, comorbidity score,^22^^,^^23^ claims-based frailty score,^24^ metabolic comorbidities^25^ (eg, overweight, obesity, and morbid obesity), diabetes-related variables, cardiovascular comorbidities, renal comorbidities, other comorbidities, medication use (eg, glucose-lowering medications, diuretics, and anti-hypertensive medications), and health care utilization metrics (eg, specialist visits and emergency department visits).

### Statistical Analysis

To address potential confounding, propensity score matching was implemented separately for both cohorts (incident NPDR and DR progression). Propensity scores estimating the likelihood of initiating an SGLT2i versus GLP1RA were derived from multivariable logistic regression models that included 103 predetermined covariates. Patients were matched 1:1 using nearest neighbor matching with a 0.02 caliper.^26^ Covariate balance was evaluated via standardized differences, with values under 0.1 indicating satisfactory balance.^27^ Means were reported with standard deviations (SDs) and medians with interquartile ranges (IQRs). Matched cohorts were analyzed for incidence rates (IRs) per 1,000 person-years, rate differences (RDs), and hazard ratios (HRs) with 95% confidence intervals (CIs). IR differences were calculated through generalized linear regression with an identity link and normal error distribution.^28^ Cox proportional hazards models were used to calculate HRs and 95% CIs. Results were pooled across the three databases using fixed-effects meta-analysis. We generated cumulative incidence curves and calculated log-rank tests to compare event rates between the SGLT2i and GLP1RA groups. All analyses were conducted using the Aetion Evidence Platform software (version 5.18.2, Aetion, Inc) for real-world data analysis,^29^^,^^30^ and the RStudio software (version 2023.06.0+421, Posit Software, PBC).

### Subgroup and Sensitivity Analyses

Robustness of the primary results was evaluated through subgroup and sensitivity analyses. Subgroups were examined based on age (<70 vs. ≥70 years), sex, smoking status, hypertension, diabetic neuropathy, recent insulin use (prior 90 days until cohort entry date), uncontrolled diabetes status (defined by hyperglycemia, diabetic ketoacidosis, or hyperosmolar hyperglycemic nonketotic syndrome), proteinuria, and cohort entry year (2014-2019 vs 2020-2024). Propensity scores were recalculated within each subgroup, with matching methods consistent with the primary analyses.^31^ We also performed sensitivity analyses by changing the drug discontinuation grace period from 60 days to 30 days and by conducting intention-to-treat analyses. The latter did not censor at index treatment discontinuation or switching in order to address potential informative censoring.

## Results

### Incident NPDR Cohort

From an initial cohort of 4,544,444 patients aged ≥18 years who initiated either an SGLT2i or GLP1RA, 4,458,737 patients were excluded, resulting in 85,707 eligible patients (37,824 patients initiated SGLT2i and 42,575 patients initiated GLP1RA, (Fig 1A). After 1:1 propensity score matching, there were 20,135 patients in each treatment arm (SGLT2i vs GLP1RA).

**Figure 1:**
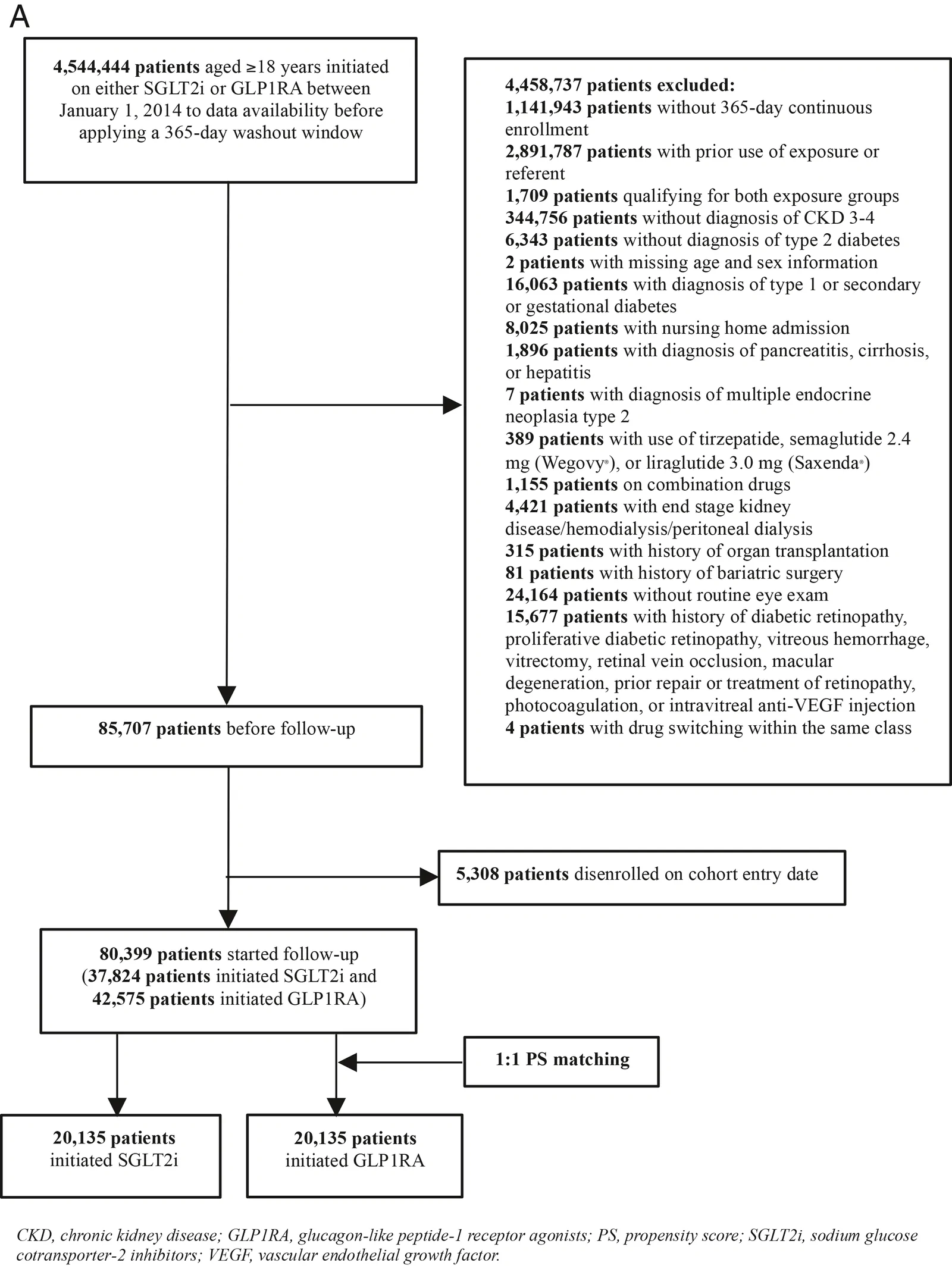

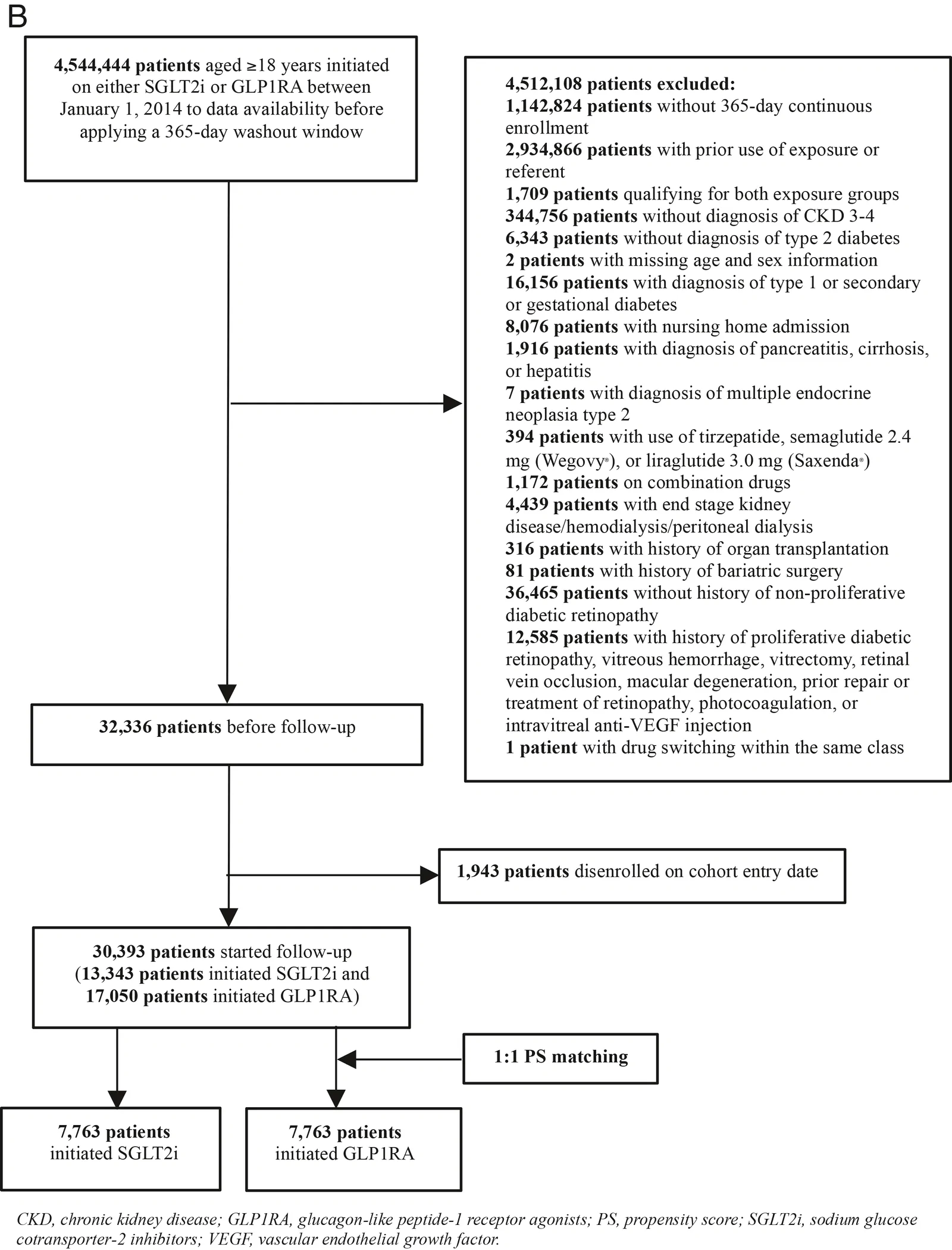
(A) Flowchart of included patients in the incident non-proliferative diabetic retinopathy cohort. (B) Flowchart of included patients in the diabetic retinopathy progression cohort.

After 1:1 PS matching, mean age was 71.78 years in the SGLT2i group and 71.74 years in the GLP1RA group, with females comprising 50% of each group. About 25% and 22% of included patients in SGLT2i and GLP1RA groups had obesity and morbid obesity, respectively. Up to 25% of patients had a history of smoking on index date. Hypertension was highly prevalent (94.6% in both groups), and 86.9% of patients in both groups had hyperlipidemia. Baseline cardiovascular disease was identified in 51% of both groups. Approximately 29.7% of patients in each group used insulin. The mean HbA1c levels were 7.64% for SGLT2i and 7.77% for GLP1RA initiators. The distribution of GLP1RA use was 38.0% semaglutide, 35.0% dulaglutide, 18.7% liraglutide, 7.6% exenatide, and 0.7% albiglutide. At baseline, glucose-lowering medications that were used included metformin (60.8% in SGLT2i and 61.0% in GLP1RA) and sulfonylureas (44.7% in SGLT2i and 44.4% in GLP1RA). About 84.8% and 84.7% of patients used statins in the SGLT2i and GLP1RA groups, respectively. The baseline characteristics of the Incident NPDR cohort is shown in Table 1 and Table S3 (full table).

**Table 1:** Truncated Baseline Characteristics of Included Patients Before and After 1:1 Propensity Score Matching

Characteristics	Incident NPDR Cohort						DR Progression Cohort					
	Before 1:1 PS Matching			After 1:1 PS Matching			Before 1:1 PS Matching			After 1:1 PS Matching		
	SGLT2i	GLP1RA	SMD	SGLT2i	GLP1RA	SMD	SGLT2i	GLP1RA	SMD	SGLT2i	GLP1RA	SMD
**Number of patients**	37,824	42,575		20,135	20,135		13,343	17,050		7,763	7,763	
**Demographics**												
Mean age ± SD	73.36 ± 7.18	71.58 ± 6.80	−0.25	71.78 ± 7.36	71.74 ± 7.21	−0.01	73.09 ± 7.42	71.89 ± 6.94	−0.17	71.86 ± 7.56	71.84 ± 7.45	0.00
Female; n (%)	16,663 (44.1%)	22,207 (52.2%)	0.16	10,016 (49.7%)	10,024 (49.8%)	0.00	6,025 (45.2%)	8,889 (52.1%)	0.14	3,790 (48.8%)	3,810 (49.1%)	0.01
Mean combined comorbidity score ± SD	3.55 ± 2.72	3.40 ± 2.47	−0.06	3.36 ± 2.57	3.39 ± 2.55	0.01	3.74 ± 2.77	3.69 ± 2.55	−0.02	3.62 ± 2.63	3.62 ± 2.64	0.00
Frailty score categories:												
0.00-0.14; n (%)	7,582 (20.0%)	6,137 (14.4%)	−0.15	4,647 (23.1%)	4,650 (23.1%)	0.00	2,559 (19.2%)	2,167 (12.7%)	−0.18	1,636 (21.1%)	1,599 (20.6%)	−0.01
0.15-0.24; n (%)	21,412 (56.6%)	23,084 (54.2%)	−0.05	11,286 (56.1%)	11,225 (55.7%)	−0.01	7,320 (54.9%)	8,736 (51.2%)	−0.07	4,247 (54.7%)	4,250 (54.7%)	0.00
≥0.25; n (%)	8,830 (23.3%)	13,354 (31.4%)	0.18	4,202 (20.9%)	4,260 (21.2%)	0.01	3,464 (26.0%)	6,147 (36.1%)	0.22	1,880 (24.2%)	1,914 (24.7%)	0.01
**Metabolic comorbidities:**												
Overweight (BMI 25-29.9 kg/m^2^); n (%)	5,125 (13.5%)	4,363 (10.2%)	−0.10	2,293 (11.4%)	2,316 (11.5%)	0.00	1,857 (13.9%)	1,828 (10.7%)	−0.10	950 (12.2%)	941 (12.1%)	0.00
Obesity (BMI 30-39.9 kg/m^2^); n (%)	8,680 (22.9%)	11,179 (26.3%)	0.08	5,134 (25.5%)	5,091 (25.3%)	0.00	2,983 (22.4%)	4,416 (25.9%)	0.08	1,866 (24.0%)	1,888 (24.3%)	0.01
Morbid obesity (BMI ≥40 kg/m^2^); n (%)	6,265 (16.6%)	11,141 (26.2%)	0.24	4,429 (22.0%)	4,556 (22.6%)	0.01	2,277 (17.1%)	4,291 (25.2%)	0.20	1,635 (21.1%)	1,654 (21.3%)	0.00
Smoking; n (%)	9,802 (25.9%)	11,214 (26.3%)	0.01	4,949 (24.6%)	4,975 (24.7%)	0.00	3,257 (24.4%)	4,358 (25.6%)	0.03	1,848 (23.8%)	1,851 (23.8%)	0.00
NPDR severities:												
Mild; n (%)	-	-	-	-	-	-	6,145 (46.1%)	8,282 (48.6%)	0.05	3,639 (46.9%)	3,712 (47.8%)	0.02
Moderate; n (%)	-	-	-	-	-	-	1,420 (10.6%)	1,930 (11.3%)	0.02	918 (11.8%)	836 (10.8%)	−0.03
Severe; n (%)	-	-	-	-	-	-	247 (1.9%)	317 (1.9%)	0.00	172 (2.2%)	146 (1.9%)	−0.02
**Diabetes-related covariates:**												
Mean HbA1c level ± SD	7.64 ± 1.47	7.77 ± 1.56	0.09	7.64 ± 1.47	7.77 ± 1.56	0.09	7.64 ± 1.47	7.77 ± 1.56	0.09	7.64 ± 1.47	7.77 ± 1.56	0.09
Insulin use; n (%)	8,160 (21.6%)	17,034 (40.0%)	0.41	5,979 (29.7%)	5,985 (29.7%)	0.00	5,668 (42.5%)	10,629 (62.3%)	0.40	4,093 (52.7%)	4,120 (53.1%)	0.01
Metformin use; n (%)	22,957 (60.7%)	23,048 (54.1%)	−0.13	12,234 (60.8%)	12,284 (61.0%)	0.00	7,970 (59.7%)	8,448 (49.5%)	−0.21	4,428 (57.0%)	4,445 (57.3%)	0.01
Sulfonylurea use; n (%)	15,588 (41.2%)	18,832 (44.2%)	0.06	9,006 (44.7%)	8,942 (44.4%)	−0.01	5,904 (44.2%)	6,877 (40.3%)	−0.08	3,327 (42.9%)	3,306 (42.6%)	−0.01
Thiazolidinedione use; n (%)	3,887 (10.3%)	4,467 (10.5%)	0.01	2,340 (11.6%)	2,303 (11.4%)	−0.01	1,682 (12.6%)	1,957 (11.5%)	−0.03	1,009 (13.0%)	1,016 (13.1%)	0.00
DPP4i use; n (%)	9,326 (24.7%)	11,200 (26.3%)	0.04	5,055 (25.1%)	5,032 (25.0%)	0.00	3,406 (25.5%)	4,262 (25.0%)	−0.01	1,925 (24.8%)	1,894 (24.4%)	0.01
**Renal comorbidities:**												
Proteinuria; n (%)^a^	5,617 (14.9%)	6,218 (14.6%)	−0.01	2,753 (13.7%)	2,780 (13.8%)	0.00	2,471 (18.5%)	3,064 (18.0%)	−0.01	1,373 (17.7%)	1,356 (17.5%)	−0.01
**Cardiovascular comorbidities:**												
Hypertension; n (%)	36,038 (95.3%)	40,664 (95.5%)	0.01	19,043 (94.6%)	19,056 (94.6%)	0.00	12,726 (95.4%)	16,361 (96.0%)	0.03	7,406 (95.4%)	7,394 (95.2%)	−0.01
Hyperlipidemia; n (%)	33,019 (87.3%)	37,232 (87.5%)	0.01	17,489 (86.9%)	17,507 (86.9%)	0.00	11,495 (86.2%)	14,711 (86.3%)	0.00	6,668 (85.9%)	6,663 (85.8%)	0.00
Baseline cardiovascular disease; n (%)^b^	21,613 (57.1%)	21,952 (51.6%)	−0.11	10,280 (51.1%)	10,269 (51.0%)	0.00	8,141 (61.0%)	9,978 (58.5%)	−0.05	4,440 (57.2%)	4,415 (56.9%)	−0.01
**Medication utilization:**												
Medications to reduce IOP; n (%)	4,898 (12.9%)	4,688 (11.0%)	−0.06	2,434 (12.1%)	2,222 (11.0%)	−0.03	1,315 (9.9%)	1,572 (9.2%)	−0.02	786 (10.1%)	713 (9.2%)	−0.03
ACEi and ARBs; n (%)	30,221 (79.9%)	33,721 (79.2%)	−0.02	16,209 (80.5%)	16,209 (80.5%)	0.00	10,906 (81.7%)	13,796 (80.9%)	−0.02	6,342 (81.7%)	6,352 (81.8%)	0.00
β Blockers; n (%)	22,166 (58.6%)	23,559 (55.3%)	−0.07	10,997 (54.6%)	10,988 (54.6%)	0.00	8,248 (61.8%)	10,465 (61.4%)	−0.01	4,639 (59.8%)	4,660 (60.0%)	0.00
Calcium channel blockers; n (%)	16,616 (43.9%)	17,710 (41.6%)	−0.05	8,656 (43.0%)	8,597 (42.7%)	−0.01	6,382 (47.8%)	8,001 (46.9%)	−0.02	3,681 (47.4%)	3,621 (46.6%)	−0.02
Antianginal agents; n (%)	4,745 (12.5%)	4,600 (10.8%)	−0.05	2,146 (10.7%)	2,166 (10.8%)	0.00	1,959 (14.7%)	2,448 (14.4%)	−0.01	1,013 (13.0%)	1,051 (13.5%)	0.01
**Health care utilization:**												
Mean internist visits ± SD^c^	13.76 ± 31.80	13.22 ± 28.30	−0.02	14.46 ± 32.43	14.46 ± 31.58	0.00	12.73 ± 33.45	12.21 ± 29.40	−0.02	12.86 ± 33.50	12.86 ± 34.68	0.00
Mean endocrinologist visits ± SD^c^	1.25 ± 5.50	2.13 ± 7.00	0.14	1.49 ± 5.95	1.49 ± 5.51	0.00	1.51 ± 5.54	2.35 ± 6.75	0.14	1.73 ± 6.02	1.80 ± 5.59	0.01
Mean cardiologist visits ± SD^c^	5.36 ± 10.13	4.29 ± 8.64	−0.11	4.05 ± 7.97	4.14 ± 8.36	0.01	5.28 ± 9.89	4.67 ± 9.09	−0.06	4.46 ± 8.78	4.46 ± 8.78	0.00
Mean nephrologist visits ± SD^c^	1.33 ± 4.18	1.57 ± 4.72	0.05	1.31 ± 4.31	1.31 ± 3.91	0.00	1.45 ± 4.99	1.73 ± 5.25	0.05	1.50 ± 5.56	1.51 ± 4.68	0.00
Mean hospitalization events ± SD	0.24 ± 0.66	0.22 ± 0.60	−0.03	0.19 ± 0.57	0.19 ± 0.57	0.00	0.28 ± 0.69	0.27 ± 0.67	−0.01	0.24 ± 0.62	0.24 ± 0.64	0.00
Mean emergency department visits ± SD	0.65 ± 1.61	0.71 ± 1.68	0.04	0.58 ± 1.44	0.59 ± 1.45	0.01	0.70 ± 1.56	0.82 ± 1.76	0.07	0.67 ± 1.57	0.66 ± 1.58	−0.01

Abbreviations: ACEi, angiotensin converting enzyme inhibitors; ARB, angiotensin receptor blockers; BMI, body mass index; DR, diabetic retinopathy; ED, emergency department; GLP1RA, glucagon-like peptide-1 receptor agonist; IOP, intraocular pressure: NPDR, non-proliferative diabetic retinopathy; SD, standard deviation; SMD, standard mean difference; SGLT2i, sodium-glucose cotransporter-2 inhibitor.

a Proteinuria variable is defined by ICD codes, not by individual urine protein-creatinine ratio or urine microalbumin-creatinine ratio.

b Includes stable angina, unstable angina, acute myocardial infarctions, sequalae of myocardial infarction, coronary atherosclerosis, coronary procedure, heart failure, ischemic stroke, and peripheral artery disease.

c Only outpatient visits.

Over a median (IQR) follow-up of 200 (106-408) days, incident NPDR developed in 524 patients (29.55 per 1,000 person-years) in the SGLT2i group and 543 patients (30.38 per 1,000 person-years) in the GLP1RA group, with an RD of −0.83 per 1,000 PY (95% CI, −4.43 to 2.76) and an HR of 0.98 (95% CI, 0.87-1.10) (Table 2). The cumulative incidence curves demonstrated no difference in the risk of incident NPDR between the SGLT2i and GLP1RA groups over the 24-month follow-up period (log-rank test *P* = 0.6; Fig 2A). Censoring reasons for incident NPDR are shown in Table S4. More than 50% (∼51%-57%) of patients in either the SGLT2i or GLP1RA group were censored due to the end of index exposure, followed by the end of patient data (18%-20%).

**Table 2:** Analysis of Incident Non-Proliferative Diabetic Retinopathy and Diabetic Retinopathy Progression Outcomes Between SGLT2i Versus GLP1RA Initiators After 1:1 Propensity Score Matching

Incident Non-proliferative Diabetic Retinopathy								
Database	SGLT2i			GLP1RA			Measures of Association	
	No. of Subjects	Events	IR/1,000 PY (95% CI)	No. of Subjects	Events	IR/1,000 PY (95% CI)	RD/1,000 PY (95% CI)	HR (95% CI)
**Medicare**	6,385	159	30.15 (25.65-35.22)	6,385	183	32.15 (27.66-37.16)	−2.00 (−8.60 to 4.61)	0.94 (0.76-1.16)
**MarketScan**	3,312	81	30.94 (24.57-38.46)	3,312	80	27.97 (22.18-34.81)	2.97 (−6.14 to 12.08)	1.12 (0.82-1.53)
**Optum CDM**	10,438	284	28.85 (25.59-32.41)	10,438	280	30.04 (26.62-33.77)	−1.19 (−6.05 to 3.68)	0.96 (0.82-1.13)
**Pooled**	20,135	524	29.55 (27.07-32.19)	20,135	543	30.38 (27.88-33.05)	−0.83 (−4.43 to 2.76)	0.98 (0.87-1.10)

Diabetic retinopathy progression								
Database	SGLT2i			GLP1RA			Measures of association	
	No. of subjects	Events	IR/1,000 PY (95% CI)	No. of subjects	Events	IR/1,000 PY (95% CI)	RD/1,000 PY (95% CI)	HR (95% CI)
**Medicare**	2,692	56	27.68 (20.91-35.95)	2,692	62	30.10 (23.08-38.58)	−2.42 (−12.84 to 8.01)	0.91 (0.64-1,31)
**MarketScan**	1,173	36	42.70 (29.91-59.12)	1,173	40	43.57 (31.13-59.33)	−0.87 (−20.28 to 18.55)	0.96 (0.61-1.50)
**Optum CDM**	3,898	109	33.27 (27.32-40.14)	3,898	118	36.14 (29.91-43.28)	−2.87 (−11.90 to 6.16)	0.92 (0.71-1.19)
**Pooled**	7,763	201	32.73 (28.36-37.58)	7,763	220	35.24 (30.74-40.22)	−2.51 (−9.01 to 3.98)	0.92 (0.76-1.12)

Abbreviations: CI, confidence interval; GLP1RA, glucagon-like peptide-1 receptor agonists; HR, hazard ratio; IR, incidence rate; Optum CDM, Optum’s de-identified Clinformatics Data Mart; PY, person-year; RD, rate difference; SGLT2i, sodium-glucose cotransporter-2 inhibitors.

**Figure 2:**
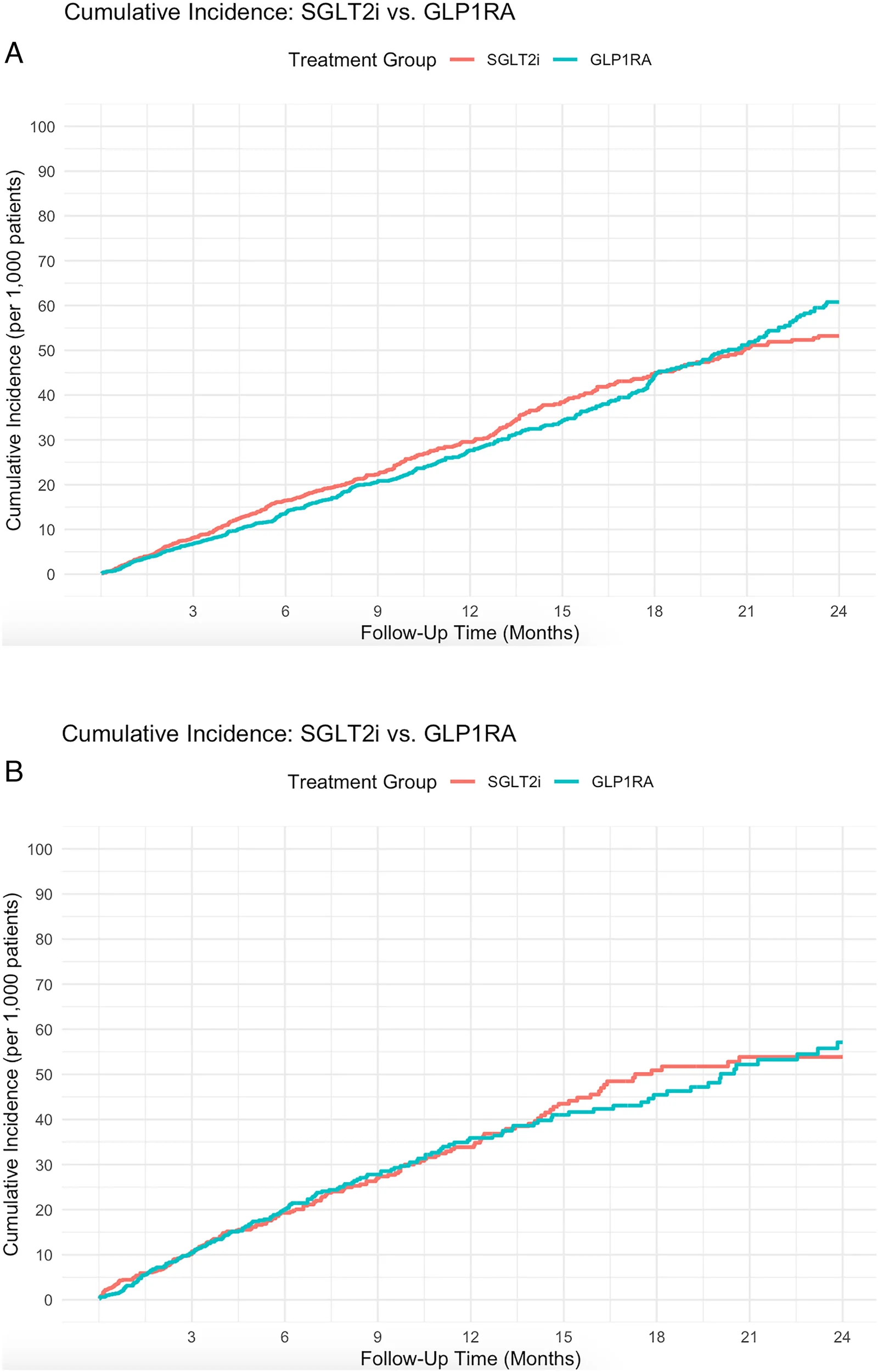
(A) Cumulative incidence (per 1,000 patients) of incident non-proliferative diabetic retinopathy outcome comparing SGLT2i and GLP1RA initiators. GLP1RA, glucagon-like peptide-1 receptor agonists; SGLT2i, sodium-glucose cotransporter-2 inhibitors.B) Cumulative incidence (per 1,000 patients) of diabetic retinopathy progression outcome comparing SGLT2i and GLP1RA initiators. GLP1RA, glucagon-like peptide-1 receptor agonists; SGLT2i, sodium-glucose cotransporter-2 inhibitors.

Subgroup analyses evaluating incident NPDR outcomes comparing SGLT2i and GLP1RA initiators showed no differences in HRs across multiple predefined subgroups, including age, sex, smoking history, hypertension, diabetic neuropathy, insulin use, uncontrolled diabetes, proteinuria, and cohort entry year (all *P*-interaction > 0.05; Fig 3A). Sensitivity analyses further supported the robustness of these findings; results remained consistent when the drug discontinuation grace period was shortened to 30 days (HR 1.01; 95% CI, 0.88-1.15) and in an intention-to-treat analysis (HR 1.05; 95% CI, 0.93-1.19) (Table S5).

**Figure 3:**
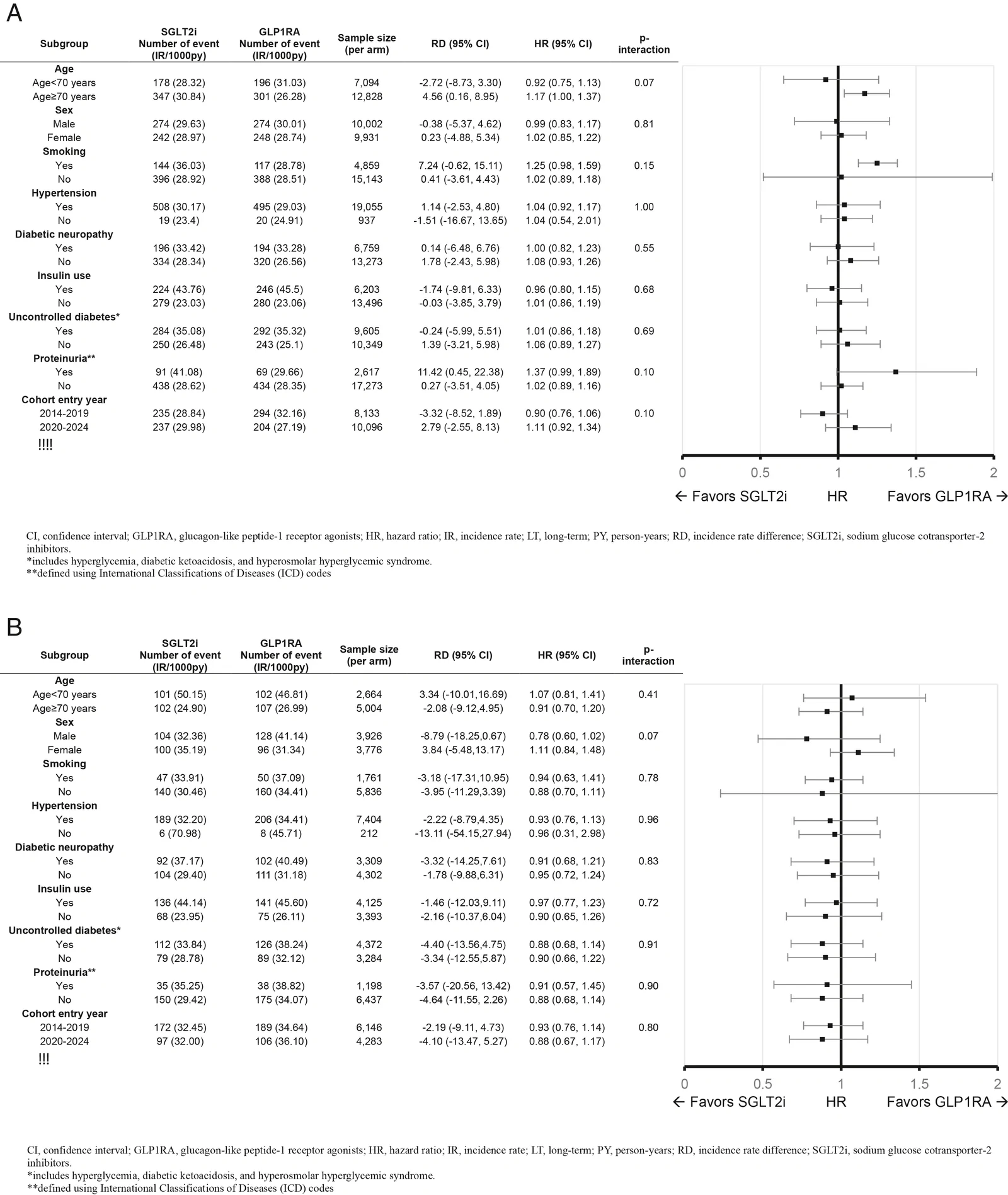
Subgroup analysis of outcomes comparing SGLT2i and GLP1RA initiators. GLP1RA, glucagon-like peptide-1 receptor agonists; SGLT2i, sodium-glucose cotransporter-2 inhibitors.

### DR Progression Cohort

From an initial cohort of 4,544,444 patients aged ≥18 years who initiated either an SGLT2i or GLP1RA, 4,512,108 patients were excluded, leaving 32,336 eligible patients (13,343 patients initiated SGLT2i and 17,050 patients initiated GLP1RA) (Fig 1B). After 1:1 propensity score matching, 7,763 patients were included in each of the SGLT2i and GLP1RA treatment groups.

After 1:1 PS matching, the mean age was 71.86 years in the SGLT2i group, and 71.84 years in the GLP1RA group, with females comprising ∼49% in both groups. Obesity and morbid obesity were present in ∼24% and 21% of patients in each group, respectively. Smoking history was identified in about 24% of patients. Around 47%, and 11% of patients had mild and moderate NPDR, respectively. Hypertension was highly prevalent, affecting 95.4% in the SGLT2i group, and 95.2% in the GLP1RA group. Hyperlipidemia was present in ∼86% of patients in both groups, and baseline cardiovascular disease was identified in about 57% of each group. A higher proportion of patients in this cohort used insulin—52.7% in the SGLT2i group and 53.1% in the GLP1RA group. The mean HbA1c levels were 7.64% in the SGLT2i group and 7.77% in the GLP1RA group. The distribution of GLP1RA was 39.8% dulaglutide, 35.7% semaglutide, 17.9% liraglutide, 6.3% exenatide, and 0.4% albiglutide. Baseline glucose-lowering medications that were used included metformin (57.0% in SGLT2i, and 57.3% in GLP1RA) and sulfonylureas (42.9% in SGLT2i and 42.6% in GLP1RA). Statin use was similar between both groups (87% in each group). Detailed baseline characteristics of the DR progression cohort are provided in Table 1 and Table S3 (full table).

Over a median (IQR) follow-up of 175 (101-364) days, DR progression occurred in 201 patients (32.73 per 1,000 person-years) in the SGLT2i group and 220 patients (35.24 per 1,000 person-years) in the GLP1RA group, corresponding to a RD of −2.51 per 1,000 PY (95% CI, −9.01 to 3.98), and a HR of 0.92 (95% CI, 0.76-1.12) and (Table 2). The cumulative incidence curves indicated no difference in the risk of DR progression between the SGLT2i and GLP1RA groups over the 24-month follow-up period (log-rank test *P* = 0.4; Fig 2B). Censoring reasons for DR progression are shown in Table S4. More than 50% (∼51%-57%) of patients in either SGLT2i or GLP1RA group were censored due to end of index exposure, followed by end of patient data (18%-21%).

Subgroup analyses evaluating DR progression comparing SGLT2i versus GLP1RA initiators revealed no differences in HRs across multiple predefined subgroups, including age, sex, smoking history, hypertension, diabetic neuropathy, insulin use, uncontrolled diabetes, proteinuria, and cohort entry year (all *P*-interaction > 0.05; Fig 3B). Sensitivity analyses reinforced the robustness of these findings, demonstrating consistent hazard ratios when the drug discontinuation grace period was shortened to 30 days (HR 0.97; 95% CI, 0.79-1.20) and in intention-to-treat analyses (HR 0.93; 95% CI, 0.78-1.12) (Table S5).

## Discussion

In this real-world study of patients with CKD stages 3-4 and T2D comparing SGLT2i versus GLP1RA initiators, we found no difference between groups in the risk of incident NPDR or DR progression over a median follow-up of 6-7 months. Subgroup and sensitivity analyses supported the robustness of our primary findings.

Current evidence regarding the effects of SGLT2i on retinopathy outcomes remains limited. Our results are consistent with a post hoc analysis of the placebo-controlled EMPA-REG outcome trial, in which retinopathy outcomes did not differ between empagliflozin and placebo groups (HR 0.78 [95% CI 0.54-1.12]; *P* = 0.17).^13^ Notably, however, only 25% of EMPA-REG participants had CKD (defined by estimated glomerular filtration rate [GFR] <60 mL/min/1.73m^2^), and just 10% exhibited albuminuria (>300 mg/g).^32^ Similarly, another real-world study reported comparable incident NPDR risk between empagliflozin and dipeptidyl peptidase-4 inhibitors, although SGLT2i showed a 22% reduced risk for DR progression compared to dipeptidyl peptidase-4 inhibitors. However, subgroup analyses of patients with CKD were not conducted.^14^ Additionally, a recent meta-analysis of randomized controlled trials observed no significant association between SGLT2i use and DR incidence overall but noted a lower risk among patients with shorter diabetes duration (<10 years).^33^

In contrast, the trial to evaluate cardiovascular and other long-term outcomes with semaglutide in subjects with type 2 diabetes (SUSTAIN-6) reported a 76% increased risk of retinopathy complications (eg, vitreous hemorrhage, blindness, or treatments requiring intravitreal agents or photocoagulation) with semaglutide compared to placebo (IR 1.49 vs 0.86 events/100 patient-years, respectively; HR 1.76 [95% CI 1.11-2.78]; *P* = 0.02]).^16^ Approximately 30% of participants in the SUSTAIN-6 trial had CKD (eGFR <60 mL/min/1.73m^2^). The underlying mechanism for this increased risk remains uncertain but is hypothesized to result from rapid glucose lowering within three months among people with proliferative DR at baseline.^34^ Conversely, the LEADER trial involving liraglutide found no significant difference in retinopathy outcomes compared to placebo (HR 1.15 [95% CI 0.87-1.52]; *P* = 0.33).^35^ These conflicting findings suggest that retinopathy outcomes might differ across baseline risk factors or individual GLP1RA agents related to their potency of glucose lowering, which may partly explain discrepancies with our study, as we included multiple GLP1RA agents available in clinical practice rather than semaglutide alone.

Sodium-glucose cotransporter-2 is primarily lack direct mechanisms targeting the retinal microvasculature. SGLT2 receptors are not abundantly expressed in retinal tissue.^36^ This means that SGLT2i do not have a strong direct site of action within the retinal cells.^36^ Moreover, the retina’s specialized blood-retinal barrier tightly regulates molecular exchange, limiting penetration of systemic therapies such as SGLT2i.^37^ Furthermore, the effect of glycemic control from SGLT2i is considered modest compared to other glucose-lowering medications, especially in patients with CKD, in which the glucose-lowering effect is blunted as eGFR declines.^38^ The net result is that SGLT2i neither dramatically reduces intra-retinal hyperglycemia nor actively engages retinal protective pathways, beyond what is achieved by modest glycemic control.

We acknowledge several limitations to our study. First, residual or unmeasured confounding cannot be excluded. For example, we lacked information on diabetes duration, an important risk factor for DR, individual SGLT2i or GLP1RA agent breakdown, and some laboratory values. Nonetheless, our cohorts are well matched to minimize the risk of residual confounding.^39^ Second, CKD staging was ascertained using diagnosis codes only. However, the codes used in our study have shown high specificity for CKD in previous validation studies.^18^^,^^40^^,^^41^ Similarly, claims data on DR lack clinical granularity eg, we cannot ascertain the clinical findings for the definitions of NPDR severity or obtain Early Treatment Diabetic Retinopathy Study (ETDRS) measures. Fourth, outcome misclassification based on DR diagnosis codes from claims databases may have occurred, potentially missing certain progression events such as mild to moderate NPDR transitions. Nevertheless, misclassification is likely nondifferential between the exposure groups. Fifth, our study was designed to specifically address the incidence of NPDR among patients without a history of any DR and DR progression among patients with NPDR irrespective of diabetic macular edema, which could develop at any stage of retinopathy. The diabetic macular edema endpoint would require a new cohort definition, which will be available in our future study. Finally, although the median follow-up in our study was relatively short (∼6-7 months), reflecting real-world medication adherence patterns, a longer follow-up duration is recommended given the timeframe typically required for DR to develop and progress. Notably, when worsening retinopathy results from rapid intensification of glycemic control, it can occur within a short or longer time frame but is called early worsening because it can be seen as early as 3 months.^42^

In conclusion, among real-world patients with CKD stages 3-4 and T2D, SGLT2i was not associated with a different risk of incident NPDR or DR progression when compared with GLP1RA after a relatively short observation period of 6-7 months. Future studies with longer follow-up durations are warranted to fully characterize the long-term retinal safety and efficacy profiles of these therapies.

For practicing nephrologists, these findings suggest that DR risk may not be a major short-term differentiator when choosing between SGLT2i and GLP1RA in patients with CKD stages 3-4 and T2D. In most cases, treatment selection should continue to prioritize kidney and cardiovascular indications (rather than DR as an indication), glycemic needs, weight effects, tolerability, route of administration, and access. In patients with established retinopathy, especially when rapid glycemic improvement is anticipated, closer ophthalmologic follow-up remains appropriate.
