# Supplementary material for: Diabetic Retinopathy Risk in SGLT2i vs GLP1RA Initiators With CKD Stages 3-4 and Type 2 Diabetes
Source: Kidney Med. 2026 Sep 9;8(11):101518. doi: 10.1016/j.xkme.2026.101518 (PMC13697828; doi:10.1016/j.xkme.2026.101518)
Supplement: Supplementary file 1 [file mmc1.pdf]

Table S1. Definitions of key inclusion and exclusion criteria.

Inclusion/exclusion criteria	Codes	Assessment period [day]	Care Setting	Position
Chronic kidney disease stages 3-4	ICD-9 DX: 585.3, 585.4<br><br>ICD-10 DX: N18.3x, N18.4	[-365, 0]	Inpatient, Outpatient, Carrier Claims	Any
Diabetes type 2	ICD-9 DX: 250.x0, 250.x2<br><br>ICD-10 DX: E11.xxxx	[-365, 0]	Inpatient, Outpatient, Carrier Claims	Any
Retinal or vision exam or provider code [applied only to Incident NPDR cohort]	Routine retinal or vision exam: HCPCS: 92002, 92003, 92004, 92012, 92014, 92018, 92019, 3072F, 92227, 92228, G8397<br><br>Ophthalmologist or Optometrist visit identified using Provider Specialty Code	[-365, 0]	Inpatient, Outpatient, Carrier Claims	Any
History of NPDR [applied only to DR progression cohort]	ICD-9 DX: 362.04, 362.05, 362.06, 362.03<br><br>ICD-10 DX: E10.321, E10.3212, E10.3213, E10.3219, E10.3291, E10.3292, E10.3311, E10.3313, E10.3391, E10.3393, E10.341, E10.3411, E10.3491, E10.3492, E10.3499, E11.3211, E11.3213, E11.3219, E11.329, E11.3291, E11.331, E11.3311, E11.3312, E11.3391, E11.3393, E11.3411, E11.3491, E13.321, E13.3219, E13.329, E13.3291, E13.3299, E13.3319, E13.34, E13.341, E13.3411, E13.349, E13.3492, E13.3493, E13.3499, E10.3211, E10.329, E10.3293, E10.3299, E10.331, E10.3312, E10.3319, E10.339, E10.3392, E10.3399, E10.3412, E10.3413, E10.3419, E10.349, E10.3493, E11.321, E11.3212, E11.3292, E11.3293, E11.3299, E11.3313, E11.3319, E11.339, E11.3392, E11.3399, E11.341, E11.3412, E11.3413, E11.3419, E11.349, E11.3492, E11.3493, E11.3499, E13.32, E13.3211, E13.3212, E13.3213, E13.3292, E13.3293, E13.331, E13.3311, E13.3312, E13.3313, E13.3412, E13.3413, E13.3419, E13.3491	[since all available data, 0]	Inpatient, Outpatient, Carrier Claims	Any
Diabetes type 1	ICD-9 DX: 250.x1, 250.x3<br><br>ICD-10 DX: E10.xxxx	[-365, 0]	Inpatient, Outpatient, Carrier Claims	Any
Secondary diabetes	ICD-9 DX: 249.xx<br><br>ICD-10 DX: E08.xxxx, E09.xxxx	[-365, 0]	Inpatient, Outpatient, Carrier Claims	Any
Gestational diabetes	ICD-9 DX: 648.8x<br><br>ICD-10 DX: O24.4xx, O99.81x	[-365, 0]	Inpatient, Outpatient, Carrier Claims	Any
End-stage kidney disease or CKD stage 5	ICD-9 DX: 585.5, 585.6, 996.81, V42.0, V45.1x, V56.xx ICD-9 PX: 39.95, 54.98, 55.6x<br><br>ICD-10 DX: 0T.Y00Zx, 0T.Y10Zx, 3E1M39Z, 5A1Dx0Z, N18.5, N18.6, R88.0, T82.41xx, T82.42xx, T82.43xx, T82.49xx, T85.611x, T85.621x, T85.631x, T85.71xx, T86.1x, Y84.1, Z48.22, Z49.xx, Z91.15, Z94.0, Z99.2 CPT: 50360, 50365, 90920, 90921, 90924, 90925, 90935, 90937, 90939, 90940, 90945, 90947, 90957, 90958, 90959, 90960, 90961, 90962, 90965, 90966, 90969, 90970, 90989, 90993, 90999, 90997, 99512, 99559, 99512, G0257, G0314 , G0315, G0316, G0317, G0318 , G0319, G0322, G0323, G0326, G0327, S9335, S9339	[-365, 0]	Inpatient, Outpatient, Carrier Claims	Any
Organ transplant	ICD-9 DX: V42.1x, V42.6x, V42.7x, V42.8x (except for V42.81 or V42.82), V42.9x, V58.44, E878.0x ICD-9 PX: 33.5x, 33.6x, 37.51, 46.97, 50.5x, 52.8x, 55.6x, 996.8x (except for 996.85 or 996.88), V42.0x<br><br>ICD-10 DX: T86.1xx-T86.4xx, T86.81x, T86.85x, T86.89x,	[-365, 0]	Inpatient, Outpatient, Carrier Claims	Any

	T86.9xx, Y83.0x, Z48.2xx (except for Z48.290), Z94.0x-Z94.4x, Z94.82, Z94.83, Z94.89, Z94.9x ICD-10 PX: 02YAxxx, 0BYCxxx-0BYMxxx, 0DY5xxx, 0DY6xxx, 0DY8xxx, 0DYExxx, 0FSGxxx, 0FY0xxx, 0FYGxxx, 0TY0xxx, 0TY1xxx, 3E030Ux, 3E033Ux, 3E0J3Ux, 3E0J7Ux, 3E0J8Ux<br>CPT/HCPCS: 32851-32854, 33935, 33945, 44135, 44136, 47135, 47136, 48554, 48556, 50360, 50365, 50370, 50380			
Nursing home admission	CPT/HCPCS codes: 99301, 99302, 99303, 99311, 99312, 99313, 99315, 99316, 99379, 99380, G0066	[-365, 0]	Inpatient, Outpatient, Carrier Claims	Any
Proliferative diabetic retinopathy (PDR)	ICD-9 DX: 362.02<br><br>ICD-10 DX: E11.3513, E11.3522, E11.3539, E11.3543, E11.3549, E11.355, E11.3551, E11.3559, E11.3591, E11.3599, E11.351, E11.3511, E11.3512, E11.3519, E11.352, E11.3521, E11.3523, E11.3529, E11.353, E11.3531, E11.3532, E11.3533, E11.354, E11.3541, E11.3542, E11.3552, E11.3553, E11.359, E11.3592, E11.3593	[since all available data, 0]	Inpatient, Outpatient, Carrier Claims	Any
Age-related macular degeneration	ICD-9 DX: 362.51, 362.52, 362.57<br><br>ICD-10 DX: H35.31, H35.32, H35.36	[since all available data, 0]	Inpatient, Outpatient, Carrier Claims	Any
Central/branch retinal vein occlusion	ICD-9 DX: 362.35, 362.36<br><br>ICD-10 DX: H34.81, H34.811, H34.8110, H34.8111, H34.8112, H34.812, H34.8120, H34.8190, H34.8192, H34.83, H34.831, H34.8310, H34.8320, H34.8322, H34.833, H34.8330, H34.8331, H34.8332, H34.839, H34.8390, H34.8391, H34.8121, H34.8122, H34.813, H34.8130, H34.8131, H34.8132, H34.819, H34.8191, H34.8311, H34.8312, H34.832, H34.8321, H34.8392	[since all available data, 0]	Inpatient, Outpatient, Carrier Claims	Any
History of vitreous hemorrhage	ICD-9 DX: 379.23<br><br>ICD-10 DX: H43.1, H43.11, H43.13, H43.10, H43.12	[since all available data, 0]	Inpatient, Outpatient, Carrier Claims	Any
Pan retinal photocoagulation	HCPCS: 67228	[since all available data, 0]	Inpatient, Outpatient, Carrier Claims	Any
History of vitrectomy	HCPCS: 67036, 67040, 67041, 67042, 67043, 67039, 67113, 67229	[since all available data, 0]	Inpatient, Outpatient, Carrier Claims	Any
History of intravitreal anti-VEGF injection	CPT/HCPCS: 67028 [and within the same day] C9291, J0178, J2778, Q2046, C9257, Q5107, J9035, C9296, J9400	[since all available data, 0]	Inpatient, Outpatient, Carrier Claims	Any
Repair/treatment/destruction of extensive or progressive retinopathy	HCPCS: 67113, 67227, 67229	[since all available data, 0]	Inpatient, Outpatient, Carrier Claims	Any
Diabetic retinopathy [applied only to Incident NPDR cohort]	ICD-9 DX: 250.50, 250.51, 250.52, 250.53, 362.0x, 362.83<br><br>ICD-10 DX: H35.81, E10.311, E319, E10.321x, E10.329x, E10.331x, E10.329x, E10.331x, E1.339x, E10.41x, E10.349x, E10.351x, E10.352x, E10.353x, E10.354x, E10.355x, E10.359x, E10.37xx, E11.311, E11.319, E11.321x, E11.329x, E11.331x, E11.341x, E11.349x, E11351x, E11.352x, E11.353x, E11.354x, E11.355x, E11.359x, E11.37xx HCPCS: 67036, 67039, 67040, 67041, 67043, 67229, 67042, 67113, 67228	[since all available data, 0]	Inpatient, Outpatient, Carrier Claims	Any

Table S2. Outcome definitions.

**Incident Non-proliferative Diabetic Retinopathy (any diagnosis position)**	***In-patient, out-patient setting:***<br>ICD-9 Dx: 362.04, 362.03, 362.05, 362.06<br>ICD-10 Dx: E10.3213, E10.329, E10.3291, E10.3293, E10.331, E10.3311, E10.3313, E10.3399, E10.3412 , E10.349 , E10.3492, E10.3493, E11.3211, E11.3212, E11.331, E11.3319, E11.339, E11.3392, E11.3399, E11.3419, E11.3491, E11.3499, E13.321, E13.3211, E13.3212 , E13.329, E13.3319, E13.34, E13.341, E13.3412, E13.3413, E13.3419, E13.3492, E13.3493, E13.3499, E10.321, E10.3211, E10.3212, E10.3219, E10.3292, E10.3299, E10.3312, E10.3319, E10.339, E10.3391, E10.3392, E10.3393, E10.341, E10.3411, E10.3413, E10.3419, E10.3491, E10.3499, E11.321, E11.3213, E11.3219, E11.329, E11.3291, E11.3292, E11.3293, E11.3299, E11.3311, E11.3312, E11.3313, E11.3391, E11.3393, E11.341, E11.3411, E11.3412, E11.3413, E11.349, E11.3492, E11.3493, E13.32, E13.3213, E13.3219, E13.3291, E13.3292, E13.3293, E13.3299, E13.331, E13.3311, E13.3312, E13.3313, E13.3411, E13.349, E13.3491
**Diabetic Retinopathy Progression (any diagnosis position)**	***In-patient, out-patient setting:***<br>**Initiation of intravitreal anti-VEGF injection, or CPT/HCPCS:** 67028 [and within the same day] C9291, J0178, J2778, Q2046, C9257, Q5107, J9035, C9296, J9400<br><br>**Initiation of panretinal photocoagulation, or CPT/HCPCS:** 67228, 67210<br><br>**Onset of vitreous hemorrhage, or ICD-9 Dx:** 379.23 ICD-10 Dx: H43.1, H43.10, H43.11, H43.12, H43.13<br><br>**Onset of proliferative diabetic retinopathy:**<br>ICD-9 Dx: 362.02<br>ICD-10 Dx: E11.351, E11.3511, E11.3512, E11.3513, E11.3519, E11.352, E11.3521, E11.3522, E11.3523, E11.3529, E11.353, E11.3531, E11.3532, E11.3533, E11.3539, E11.354, E11.3541, E11.3542, E11.3543, E11.3549, E11.355, E11.3551, E11.3552, E11.3553, E11.3559, E11.359, E11.3591, E11.3592, E11.3599, E11.3593

Table S3. Baseline characteristics of included patients before and after 1:1 propensity score matching.

	Incident NPDR cohort						DR Progression cohort					
	**Before 1:1 PS matching**			**After 1:1 PS matching**			**Before 1:1 PS matching**			**After 1:1 PS matching**		
**Characteristics**	**SGLT2i**	**GLP1RA**	**SMD**	**SGLT2i**	**GLP1RA**	**SMD**	**SGLT2i**	**GLP1RA**	**SMD**	**SGLT2i**	**GLP1RA**	**SMD**
Number of patients	37824	42575		20135	20135		13343	17050		7763	7763	
**Demographics**												
Mean age (SD)	73.36 (7.18)	71.58 (6.80)	-0.25	71.78 (7.36)	71.74 (7.21)	-0.01	73.09 (7.42)	71.89 (6.94)	-0.17	71.86 (7.56)	71.84 (7.45)	0.00
Female; n (%)	16,663 (44.1%)	22,207 (52.2%)	0.16	10,016 (49.7%)	10,024 (49.8%)	0.00	6,025 (45.2%)	8,889 (52.1%)	0.14	3,790 (48.8%)	3,810 (49.1%)	0.01
Geographic region:												
...Northeast; n (%)	5,159 (13.6%)	5,740 (13.5%)	0.00	2,487 (12.4%)	2,489 (12.4%)	0.00	1,779 (13.3%)	2,432 (14.3%)	0.03	991 (12.8%)	979 (12.6%)	-0.01
...South; n (%)	16,714 (44.2%)	19,230 (45.2%)	0.02	9,262 (46.0%)	9,296 (46.2%)	0.00	5,873 (44.0%)	7,436 (43.6%)	-0.01	3,478 (44.8%)	3,482 (44.9%)	0.00
...Midwest; n (%)	7,985 (21.1%)	9,538 (22.4%)	0.03	4,227 (21.0%)	4,259 (21.2%)	0.00	2,753 (20.6%)	3,867 (22.7%)	0.05	1,656 (21.3%)	1,646 (21.2%)	0.00
...West; n (%)	7,966 (21.1%)	8,067 (18.9%)	-0.06	4,159 (20.7%)	4,091 (20.3%)	-0.01	2,938 (22.0%)	3,315 (19.4%)	-0.06	1,638 (21.1%)	1,656 (21.3%)	0.00
Race categories:												
...White; n (%)	23,945 (72.3%)	29,570 (78.8%)	0.15	12,599 (74.9%)	12,593 (74.9%)	0.00	7,544 (64.6%)	10,790 (71.4%)	0.15	4,418 (67.0%)	4,402 (66.8%)	0.00
...Black; n (%)	4,010 (12.1%)	4,030 (10.7%)	-0.04	2,009 (11.9%)	1,982 (11.8%)	0.00	1,693 (14.5%)	2,039 (13.5%)	-0.03	933 (14.2%)	949 (14.4%)	0.01
...Asian; n (%)	1,475 (4.5%)	816 (2.2%)	-0.13	480 (2.9%)	486 (2.9%)	0.00	632 (5.4%)	491 (3.2%)	-0.11	266 (4.0%)	262 (4.0%)	0.00
...Hispanic; n (%)	2,815 (8.5%)	2,023 (5.4%)	-0.12	1,413 (8.4%)	1,427 (8.5%)	0.00	1,459 (12.5%)	1,282 (8.5%)	-0.13	831 (12.6%)	815 (12.4%)	-0.01
...Other or unknown; n (%)	3,404 (10.3%)	2,801 (7.5%)	-0.10	1,633 (9.7%)	1,657 (9.8%)	0.00	1,571 (13.5%)	1,489 (9.9%)	-0.11	862 (13.1%)	882 (13.4%)	0.01
Mean combined comorbidity score (SD)	3.55 (2.72)	3.40 (2.47)	-0.06	3.36 (2.57)	3.39 (2.55)	0.01	3.74 (2.77)	3.69 (2.55)	-0.02	3.62 (2.63)	3.62 (2.64)	0.00
Frailty score categories:												
...0.00 - 0.14; n (%)	7,582 (20.0%)	6,137 (14.4%)	-0.15	4,647 (23.1%)	4,650 (23.1%)	0.00	2,559 (19.2%)	2,167 (12.7%)	-0.18	1,636 (21.1%)	1,599 (20.6%)	-0.01
...0.15 - 0.24; n (%)	21,412 (56.6%)	23,084 (54.2%)	-0.05	11,286 (56.1%)	11,225 (55.7%)	-0.01	7,320 (54.9%)	8,736 (51.2%)	-0.07	4,247 (54.7%)	4,250 (54.7%)	0.00
...>= 0.25; n (%)	8,830 (23.3%)	13,354 (31.4%)	0.18	4,202 (20.9%)	4,260 (21.2%)	0.01	3,464 (26.0%)	6,147 (36.1%)	0.22	1,880 (24.2%)	1,914 (24.7%)	0.01
**Metabolic comorbidities:**												
Underweight or normal weight (BMI < 25 kg/m^2); n (%)	1,464 (3.9%)	629 (1.5%)	-0.15	400 (2.0%)	406 (2.0%)	0.00	546 (4.1%)	336 (2.0%)	-0.12	214 (2.8%)	207 (2.7%)	-0.01
Overweight (BMI 25-29.9 kg/m^2); n (%)	5,125 (13.5%)	4,363 (10.2%)	-0.10	2,293 (11.4%)	2,316 (11.5%)	0.00	1,857 (13.9%)	1,828 (10.7%)	-0.10	950 (12.2%)	941 (12.1%)	0.00

Obesity (BMI 30-39.9 kg/m^2); n (%)	8,680 (22.9%)	11,179 (26.3%)	0.08	5,134 (25.5%)	5,091 (25.3%)	0.00	2,983 (22.4%)	4,416 (25.9%)	0.08	1,866 (24.0%)	1,888 (24.3%)	0.01
Morbid obesity (BMI ≥ 40 kg/m^2); n (%)	6,265 (16.6%)	11,141 (26.2%)	0.24	4,429 (22.0%)	4,556 (22.6%)	0.01	2,277 (17.1%)	4,291 (25.2%)	0.20	1,635 (21.1%)	1,654 (21.3%)	0.00
Smoking; n (%)	9,802 (25.9%)	11,214 (26.3%)	0.01	4,949 (24.6%)	4,975 (24.7%)	0.00	3,257 (24.4%)	4,358 (25.6%)	0.03	1,848 (23.8%)	1,851 (23.8%)	0.00
**NPDR severities:**												
...mild; n (%)	0 (0.0%)	0 (0.0%)	-	0 (0.0%)	0 (0.0%)	-	6,145 (46.1%)	8,282 (48.6%)	0.05	3,639 (46.9%)	3,712 (47.8%)	0.02
...moderate; n (%)	0 (0.0%)	0 (0.0%)	-	0 (0.0%)	0 (0.0%)	-	1,420 (10.6%)	1,930 (11.3%)	0.02	918 (11.8%)	836 (10.8%)	-0.03
...severe; n (%)	0 (0.0%)	0 (0.0%)	-	0 (0.0%)	0 (0.0%)	-	247 (1.9%)	317 (1.9%)	0.00	172 (2.2%)	146 (1.9%)	-0.02
**Diabetic comorbidities:**												
Mean HbA1c level (SD)	7.64 (1.47)	7.77 (1.56)	0.09	7.64 (1.47)	7.77 (1.56)	0.09	7.64 (1.47)	7.77 (1.56)	0.09	7.64 (1.47)	7.77 (1.56)	0.09
Hypoglycemia; n (%)	7,494 (19.8%)	8,049 (18.9%)	-0.02	4,156 (20.6%)	4,146 (20.6%)	0.00	3,183 (23.9%)	3,801 (22.3%)	-0.04	1,865 (24.0%)	1,841 (23.7%)	-0.01
Hyperglycemia; n (%)	15,869 (42.0%)	21,273 (50.0%)	0.16	9,349 (46.4%)	9,290 (46.1%)	-0.01	6,766 (50.7%)	10,063 (59.0%)	0.17	4,302 (55.4%)	4,288 (55.2%)	0.00
Diabetic ketoacidosis; n (%)	101 (0.3%)	135 (0.3%)	0.00	063 (0.3%)	061 (0.3%)	0.00	054 (0.4%)	069 (0.4%)	0.00	033 (0.4%)	030 (0.4%)	0.00
Hyperosmolar hyperglycemic nonketotic syndrome; n (%)	392 (1.0%)	478 (1.1%)	0.01	216 (1.1%)	196 (1.0%)	-0.01	132 (1.0%)	212 (1.2%)	0.02	078 (1.0%)	090 (1.2%)	0.02
Diabetic neuropathy; n (%)	11,588 (30.6%)	15,275 (35.9%)	0.11	6,671 (33.1%)	6,661 (33.1%)	0.00	5,329 (39.9%)	7,764 (45.5%)	0.11	3,340 (43.0%)	3,327 (42.9%)	0.00
Diabetic foot; n (%)	1,192 (3.2%)	1,646 (3.9%)	0.04	644 (3.2%)	658 (3.3%)	0.01	762 (5.7%)	1,154 (6.8%)	0.05	464 (6.0%)	466 (6.0%)	0.00
Infection of lower extremities; n (%)	1,960 (5.2%)	2,607 (6.1%)	0.04	1,056 (5.2%)	1,075 (5.3%)	0.00	876 (6.6%)	1,324 (7.8%)	0.05	523 (6.7%)	512 (6.6%)	0.00
Lower-limb amputations; n (%)	249 (0.7%)	339 (0.8%)	0.01	132 (0.7%)	153 (0.8%)	0.01	260 (1.9%)	372 (2.2%)	0.02	156 (2.0%)	162 (2.1%)	0.01
Median number of glucose-lowering medications (IQR):	1.60 (0.88)	2.00 (0.87)	0.46	2.00 (0.90)	2.00 (0.84)	0.00	1.60 (0.89)	2.00 (0.86)	0.46	2.00 (0.89)	2.00 (0.83)	0.00
No use of glucose-lowering medications; n (%)	4,088 (10.8%)	2,231 (5.2%)	-0.21	1,694 (8.4%)	1,819 (9.0%)	0.02	577 (4.3%)	442 (2.6%)	-0.09	332 (4.3%)	328 (4.2%)	0.00
Insulin use; n (%)	8,160 (21.6%)	17,034 (40.0%)	0.41	5,979 (29.7%)	5,985 (29.7%)	0.00	5,668 (42.5%)	10,629 (62.3%)	0.40	4,093 (52.7%)	4,120 (53.1%)	0.01
Metformin use; n (%)	22,957 (60.7%)	23,048 (54.1%)	-0.13	12,234 (60.8%)	12,284 (61.0%)	0.00	7,970 (59.7%)	8,448 (49.5%)	-0.21	4,428 (57.0%)	4,445 (57.3%)	0.01
Sulfonylurea use; n (%)	15,588 (41.2%)	18,832 (44.2%)	0.06	9,006 (44.7%)	8,942 (44.4%)	-0.01	5,904 (44.2%)	6,877 (40.3%)	-0.08	3,327 (42.9%)	3,306 (42.6%)	-0.01
Thiazolidinedione use; n (%)	3,887 (10.3%)	4,467 (10.5%)	0.01	2,340 (11.6%)	2,303 (11.4%)	-0.01	1,682 (12.6%)	1,957 (11.5%)	-0.03	1,009 (13.0%)	1,016 (13.1%)	0.00
DPP4i use; n (%)	9,326 (24.7%)	11,200 (26.3%)	0.04	5,055 (25.1%)	5,032 (25.0%)	0.00	3,406 (25.5%)	4,262 (25.0%)	-0.01	1,925 (24.8%)	1,894 (24.4%)	-0.01
Long term use of insulin; n (%)	6,897 (18.2%)	13,109 (30.8%)	0.30	4,736 (23.5%)	4,731 (23.5%)	0.00	4,691 (35.2%)	8,448 (49.5%)	0.29	3,275 (42.2%)	3,270 (42.1%)	0.00
**Renal comorbidities:**												

Acute kidney injury; n (%)	5,122 (13.5%)	5,734 (13.5%)	0.00	2,441 (12.1%)	2,489 (12.4%)	0.01	2,153 (16.1%)	2,835 (16.6%)	0.01	1,189 (15.3%)	1,220 (15.7%)	0.01
Hypertensive nephropathy; n (%)	17,223 (45.5%)	19,185 (45.1%)	-0.01	8,824 (43.8%)	8,853 (44.0%)	0.00	6,304 (47.2%)	8,228 (48.3%)	0.02	3,593 (46.3%)	3,649 (47.0%)	0.01
Proteinuria; n (%)§	5,617 (14.9%)	6,218 (14.6%)	-0.01	2,753 (13.7%)	2,780 (13.8%)	0.00	2,471 (18.5%)	3,064 (18.0%)	-0.01	1,373 (17.7%)	1,356 (17.5%)	-0.01
Miscellaneous renal disease; n (%)*	8,325 (22.0%)	10,304 (24.2%)	0.05	4,339 (21.5%)	4,387 (21.8%)	0.01	2,710 (20.3%)	3,807 (22.3%)	0.05	1,499 (19.3%)	1,512 (19.5%)	0.01
Urinary tract infection; n (%)	5,602 (14.8%)	7,936 (18.6%)	0.10	3,162 (15.7%)	3,166 (15.7%)	0.00	1,980 (14.8%)	3,081 (18.1%)	0.09	1,197 (15.4%)	1,182 (15.2%)	-0.01
Genital infections; n (%)	754 (2.0%)	1,158 (2.7%)	0.05	443 (2.2%)	470 (2.3%)	0.01	255 (1.9%)	409 (2.4%)	0.03	181 (2.3%)	164 (2.1%)	-0.01
**Cardiovascular comorbidities:**												
Hypertension; n (%)	36,038 (95.3%)	40,664 (95.5%)	0.01	19,043 (94.6%)	19,056 (94.6%)	0.00	12,726 (95.4%)	16,361 (96.0%)	0.03	7,406 (95.4%)	7,394 (95.2%)	-0.01
Hyperlipidemia; n (%)	33,019 (87.3%)	37,232 (87.5%)	0.01	17,489 (86.9%)	17,507 (86.9%)	0.00	11,495 (86.2%)	14,711 (86.3%)	0.00	6,668 (85.9%)	6,663 (85.8%)	0.00
Baseline cardiovascular disease; n (%)**	21,613 (57.1%)	21,952 (51.6%)	-0.11	10,280 (51.1%)	10,269 (51.0%)	0.00	8,141 (61.0%)	9,978 (58.5%)	-0.05	4,440 (57.2%)	4,415 (56.9%)	-0.01
Congestive heart failure; n (%)	9,714 (25.7%)	8,381 (19.7%)	-0.14	3,948 (19.6%)	3,933 (19.5%)	0.00	3,846 (28.8%)	4,284 (25.1%)	-0.08	1,890 (24.3%)	1,918 (24.7%)	0.01
Cardiomyopathy; n (%)	3,803 (10.1%)	2,527 (5.9%)	-0.16	1,260 (6.3%)	1,270 (6.3%)	0.00	1,248 (9.4%)	1,094 (6.4%)	-0.11	523 (6.7%)	541 (7.0%)	0.01
Atrial fibrillation; n (%)	7,364 (19.5%)	6,715 (15.8%)	-0.10	3,093 (15.4%)	3,105 (15.4%)	0.00	2,489 (18.7%)	2,754 (16.2%)	-0.07	1,218 (15.7%)	1,255 (16.2%)	0.01
Other cardiac dysrhythmia; n (%)	7,885 (20.8%)	7,253 (17.0%)	-0.10	3,434 (17.1%)	3,441 (17.1%)	0.00	2,654 (19.9%)	3,008 (17.6%)	-0.06	1,392 (17.9%)	1,378 (17.8%)	0.00
Valvular diseases; n (%)	7,684 (20.3%)	6,663 (15.7%)	-0.12	3,166 (15.7%)	3,211 (15.9%)	0.01	2,655 (19.9%)	2,865 (16.8%)	-0.08	1,260 (16.2%)	1,299 (16.7%)	0.01
Ischemic stroke; n (%)	5,028 (13.3%)	5,115 (12.0%)	-0.04	2,344 (11.6%)	2,359 (11.7%)	0.00	1,964 (14.7%)	2,443 (14.3%)	-0.01	1,051 (13.5%)	1,071 (13.8%)	0.01
Transient ischemic attack; n (%)	1,024 (2.7%)	1,017 (2.4%)	-0.02	477 (2.4%)	451 (2.2%)	-0.01	388 (2.9%)	510 (3.0%)	0.01	223 (2.9%)	223 (2.9%)	0.00
Peripheral artery disease; n (%)	6,130 (16.2%)	6,444 (15.1%)	-0.03	3,011 (15.0%)	2,997 (14.9%)	0.00	2,647 (19.8%)	3,379 (19.8%)	0.00	1,496 (19.3%)	1,490 (19.2%)	0.00
**Other comorbidities:**												
Asthma/Chronic obstructive pulmonary disease; n (%)	6,123 (16.2%)	6,922 (16.3%)	0.00	3,069 (15.2%)	3,093 (15.4%)	0.01	1,961 (14.7%)	2,539 (14.9%)	0.01	1,141 (14.7%)	1,110 (14.3%)	-0.01
Obstructive sleep apnea; n (%)	9,288 (24.6%)	13,052 (30.7%)	0.14	5,511 (27.4%)	5,555 (27.6%)	0.00	2,870 (21.5%)	4,533 (26.6%)	0.12	1,834 (23.6%)	1,834 (23.6%)	0.00
Gastroparesis or autonomic neuropathy; n (%)	951 (2.5%)	1,157 (2.7%)	0.01	579 (2.9%)	526 (2.6%)	-0.02	453 (3.4%)	609 (3.6%)	0.01	291 (3.7%)	251 (3.2%)	-0.03
Metabolic-associated fatty liver disease; n (%)	2,115 (5.6%)	2,693 (6.3%)	0.03	1,193 (5.9%)	1,234 (6.1%)	0.01	559 (4.2%)	767 (4.5%)	0.01	345 (4.4%)	340 (4.4%)	0.00
Hyperthyroidism; n (%)	506 (1.3%)	527 (1.2%)	-0.01	253 (1.3%)	241 (1.2%)	-0.01	162 (1.2%)	194 (1.1%)	-0.01	098 (1.3%)	075 (1.0%)	-0.03
Hypothyroidism; n (%)	9,612 (25.4%)	12,202 (28.7%)	0.07	5,269 (26.2%)	5,347 (26.6%)	0.01	3,179 (23.8%)	4,377 (25.7%)	0.04	1,872 (24.1%)	1,850 (23.8%)	-0.01
Depression; n (%)	6,036 (16.0%)	8,863 (20.8%)	0.12	3,680 (18.3%)	3,711 (18.4%)	0.00	2,019 (15.1%)	3,285 (19.3%)	0.11	1,330 (17.1%)	1,368 (17.6%)	0.01

Anxiety and sleep disorders; n (%)	8,573 (22.7%)	10,968 (25.8%)	0.07	4,833 (24.0%)	4,862 (24.1%)	0.00	2,566 (19.2%)	3,848 (22.6%)	0.08	1,611 (20.8%)	1,604 (20.7%)	0.00
Cancer; n (%)	5,002 (13.2%)	5,711 (13.4%)	0.01	2,404 (11.9%)	2,404 (11.9%)	0.00	1,503 (11.3%)	1,949 (11.4%)	0.00	799 (10.3%)	779 (10.0%)	-0.01
**Medication utilization:**												
Medications to reduce IOP; n (%)	4,898 (12.9%)	4,688 (11.0%)	-0.06	2,434 (12.1%)	2,222 (11.0%)	-0.03	1,315 (9.9%)	1,572 (9.2%)	-0.02	786 (10.1%)	713 (9.2%)	-0.03
ACEi and ARBs; n (%)	30,221 (79.9%)	33,721 (79.2%)	-0.02	16,209 (80.5%)	16,209 (80.5%)	0.00	10,906 (81.7%)	13,796 (80.9%)	-0.02	6,342 (81.7%)	6,352 (81.8%)	0.00
ARNI; n (%)	1,570 (4.2%)	375 (0.9%)	-0.21	226 (1.1%)	272 (1.4%)	0.03	525 (3.9%)	223 (1.3%)	-0.16	120 (1.5%)	127 (1.6%)	0.01
Beta blockers; n (%)	22,166 (58.6%)	23,559 (55.3%)	-0.07	10,997 (54.6%)	10,988 (54.6%)	0.00	8,248 (61.8%)	10,465 (61.4%)	-0.01	4,639 (59.8%)	4,660 (60.0%)	0.00
Calcium channel blockers; n (%)	16,616 (43.9%)	17,710 (41.6%)	-0.05	8,656 (43.0%)	8,597 (42.7%)	-0.01	6,382 (47.8%)	8,001 (46.9%)	-0.02	3,681 (47.4%)	3,621 (46.6%)	-0.02
Antianginal agents; n (%)	4,745 (12.5%)	4,600 (10.8%)	-0.05	2,146 (10.7%)	2,166 (10.8%)	0.00	1,959 (14.7%)	2,448 (14.4%)	-0.01	1,013 (13.0%)	1,051 (13.5%)	0.01
Digoxin; n (%)	971 (2.6%)	854 (2.0%)	-0.04	408 (2.0%)	364 (1.8%)	-0.01	251 (1.9%)	283 (1.7%)	-0.02	129 (1.7%)	129 (1.7%)	0.00
Antiarrhythmics; n (%)	1,874 (5.0%)	1,577 (3.7%)	-0.06	740 (3.7%)	754 (3.7%)	0.00	531 (4.0%)	602 (3.5%)	-0.03	245 (3.2%)	280 (3.6%)	0.02
Anticoagulants; n (%)	6,834 (18.1%)	6,361 (14.9%)	-0.09	2,965 (14.7%)	2,984 (14.8%)	0.00	2,264 (17.0%)	2,622 (15.4%)	-0.04	1,150 (14.8%)	1,158 (14.9%)	0.00
Antiplatelets; n (%)	6,081 (16.1%)	5,859 (13.8%)	-0.06	2,771 (13.8%)	2,844 (14.1%)	0.01	2,694 (20.2%)	3,312 (19.4%)	-0.02	1,442 (18.6%)	1,464 (18.9%)	0.01
Statins; n (%)	32,457 (85.8%)	35,414 (83.2%)	-0.07	17,083 (84.8%)	17,050 (84.7%)	0.00	11,586 (86.8%)	14,637 (85.8%)	-0.03	6,731 (86.7%)	6,720 (86.6%)	0.00
Fenofibrate; n (%)	4,296 (11.4%)	5,935 (13.9%)	0.08	2,487 (12.4%)	2,578 (12.8%)	0.01	1,185 (8.9%)	1,774 (10.4%)	0.05	738 (9.5%)	761 (9.8%)	0.01
Thiazide-like diuretics; n (%)	7,647 (20.2%)	8,930 (21.0%)	0.02	4,207 (20.9%)	4,220 (21.0%)	0.00	2,903 (21.8%)	3,880 (22.8%)	0.02	1,741 (22.4%)	1,791 (23.1%)	0.02
Loop diuretics; n (%)	11,349 (30.0%)	13,333 (31.3%)	0.03	5,580 (27.7%)	5,680 (28.2%)	0.01	4,784 (35.9%)	6,647 (39.0%)	0.06	2,698 (34.8%)	2,679 (34.5%)	-0.01
Mineralocorticoid receptor antagonists; n (%)	4,331 (11.5%)	3,961 (9.3%)	-0.07	1,850 (9.2%)	1,895 (9.4%)	0.01	1,507 (11.3%)	1,656 (9.7%)	-0.05	739 (9.5%)	767 (9.9%)	0.01
UTI antibiotics; n (%)	7,280 (19.2%)	10,198 (24.0%)	0.12	4,138 (20.6%)	4,186 (20.8%)	0.00	2,460 (18.4%)	3,925 (23.0%)	0.11	1,532 (19.7%)	1,527 (19.7%)	0.00
NSAIDs; n (%)	8,012 (21.2%)	9,327 (21.9%)	0.02	4,603 (22.9%)	4,553 (22.6%)	-0.01	2,692 (20.2%)	3,549 (20.8%)	0.01	1,666 (21.5%)	1,583 (20.4%)	-0.03
Opioids; n (%)	11,619 (30.7%)	15,917 (37.4%)	0.14	6,793 (33.7%)	6,814 (33.8%)	0.00	3,836 (28.7%)	5,981 (35.1%)	0.14	2,411 (31.1%)	2,379 (30.6%)	-0.01
Gabapentinoids; n (%)	8,270 (21.9%)	10,983 (25.8%)	0.09	4,869 (24.2%)	4,885 (24.3%)	0.00	3,351 (25.1%)	5,006 (29.4%)	0.10	2,140 (27.6%)	2,151 (27.7%)	0.00
Antidepressants; n (%)	11,481 (30.4%)	15,765 (37.0%)	0.14	6,989 (34.7%)	6,949 (34.5%)	0.00	3,695 (27.7%)	5,734 (33.6%)	0.13	2,400 (30.9%)	2,424 (31.2%)	0.01
Anxiolytics/hypnotics; n (%)	2,980 (7.9%)	3,984 (9.4%)	0.05	1,686 (8.4%)	1,790 (8.9%)	0.02	889 (6.7%)	1,235 (7.2%)	0.02	557 (7.2%)	531 (6.8%)	-0.02
Benzodiazepines; n (%)	4,664 (12.3%)	6,102 (14.3%)	0.06	2,727 (13.5%)	2,709 (13.5%)	0.00	1,272 (9.5%)	1,924 (11.3%)	0.06	827 (10.7%)	818 (10.5%)	-0.01
Antipsychotics; n (%)	1,191 (3.1%)	1,620 (3.8%)	0.04	753 (3.7%)	713 (3.5%)	-0.01	356 (2.7%)	491 (2.9%)	0.01	237 (3.1%)	211 (2.7%)	-0.02
Antiparkinsonian medications; n (%)	1,498 (4.0%)	2,192 (5.1%)	0.05	916 (4.5%)	907 (4.5%)	0.00	425 (3.2%)	674 (4.0%)	0.04	259 (3.3%)	266 (3.4%)	0.01

Dementia medications; n (%)	1,186 (3.1%)	1,093 (2.6%)	-0.03	608 (3.0%)	507 (2.5%)	-0.03	442 (3.3%)	595 (3.5%)	0.01	232 (3.0%)	260 (3.3%)	0.02
**Healthcare utilization:**												
Mean internist visits (SD)***	13.76 (31.80)	13.22 (28.30)	-0.02	14.46 (32.43)	14.46 (31.58)	0.00	12.73 (33.45)	12.21 (29.40)	-0.02	12.86 (33.50)	12.86 (34.68)	0.00
Mean endocrinologist visits (SD)***	1.25 (5.50)	2.13 (7.00)	0.14	1.49 (5.95)	1.49 (5.51)	0.00	1.51 (5.54)	2.35 (6.75)	0.14	1.73 (6.02)	1.80 (5.59)	0.01
Mean cardiologist visits (SD)***	5.36 (10.13)	4.29 (8.64)	-0.11	4.05 (7.97)	4.14 (8.36)	0.01	5.28 (9.89)	4.67 (9.09)	-0.06	4.46 (8.78)	4.46 (8.78)	0.00
Mean nephrologist visits (SD)***	1.33 (4.18)	1.57 (4.72)	0.05	1.31 (4.31)	1.31 (3.91)	0.00	1.45 (4.99)	1.73 (5.25)	0.05	1.50 (5.56)	1.51 (4.68)	0.00
Hospitalization within 60 days; n (%)	2,292 (6.1%)	1,512 (3.6%)	-0.12	888 (4.4%)	680 (3.4%)	-0.05	868 (6.5%)	745 (4.4%)	-0.09	415 (5.3%)	331 (4.3%)	-0.05
Mean hospitalization events (SD)	0.24 (0.66)	0.22 (0.60)	-0.03	0.19 (0.57)	0.19 (0.57)	0.00	0.28 (0.69)	0.27 (0.67)	-0.01	0.24 (0.62)	0.24 (0.64)	0.00
ED visit within 60 days; n (%)	3,689 (9.8%)	3,410 (8.0%)	-0.06	1,728 (8.6%)	1,451 (7.2%)	-0.05	1,340 (10.0%)	1,553 (9.1%)	-0.03	715 (9.2%)	627 (8.1%)	-0.04
Mean emergency department visits (SD)	0.65 (1.61)	0.71 (1.68)	0.04	0.58 (1.44)	0.59 (1.45)	0.01	0.70 (1.56)	0.82 (1.76)	0.07	0.67 (1.57)	0.66 (1.58)	-0.01

ACEi, angiotensin converting enzyme inhibitors; ARB, angiotensin receptor blockers; ARNI, angiotensin receptor-neprilysin inhibitor; BMI, body mass index; DR, diabetic retinopathy; ED, emergency department; GLP1RA, glucagon-like peptide-1 receptor agonist; GLM, glucose-lowering medications; IOP, intraocular pressure: NPDR, non-proliferative diabetic retinopathy; SD, standard deviation; SMD, standard mean difference; SGLT2i, sodium-glucose cotransporter-2 inhibitor.
§proteinuria variable is defined by ICD codes, not by individual urine protein-creatinine ratio or urine microalbumin-creatinine ratio.
**includes interstitial diseases, crystal-induced nephropathy, glomerular diseases, genitourinary tract abnormalities, hepatorenal syndrome, renal vascular abnormalities.*
***includes stable angina, unstable angina, acute myocardial infarctions, sequalae of myocardial infarction, coronary atherosclerosis, coronary procedure, heart failure, ischemic stroke, and peripheral artery disease.*
****only outpatient visits.*

Table S4. Censoring reasons after the start of follow-up.

**Incident Non-proliferative Diabetic Retinopathy cohort**		
**Censoring**	**SGLT2i**	**GLP1RA**
Outcome	524 (2.6%)	543 (2.7%)
Death	356 (1.8%)	353 (1.8%)
Start of an additional exposure	2,342 (11.6%)	1,560 (7.7%)
End of index exposure	10,408 (51.7%)	11,385 (56.5%)
End of patient data	3,966 (19.7%)	3,731 (18.5%)
End of patient enrollment	2,216 (11.0%)	2,157 (10.7%)
Initiation of tirzepatide, Wegovy®, or Saxenda®	323 (1.6%)	406 (2.0%)

**Diabetic Retinopathy Progression cohort**		
**Censoring**	**SGLT2i**	**GLP1RA**
Outcome	201 (2.6%)	220 (2.8%)
Death	170 (2.2%)	174 (2.2%)
Start of an additional exposure	866 (11.2%)	666 (8.6%)
End of index exposure	3,978 (51.2%)	4,403 (56.7%)
End of patient data	1,599 (20.6%)	1,445 (18.6%)
End of patient enrollment	812 (10.5%)	716 (9.2%)
Initiation of tirzepatide, Wegovy®, or Saxenda®	137 (1.8%)	139 (1.8%)

GLP1RA, glucagon-like peptide-1 receptor agonists; SGLT2i, sodium-glucose cotransporter-2 inhibitors

Table S5. Sensitivity analysis comparing SGLT2i and GLP1RA initiators.

Analysis	Incidence rate difference* (95% CI)	Hazard ratio (95% CI)
**<u>Incident NPDR cohort:</u>**		
**Primary analysis**		
60-day grace period, as treated	-0.83 (-4.43, 2.76)	0.98 (0.87, 1.10)
**Sensitivity analysis**		
30-day grace period	0.24 (-3.78, 4.26)	1.01 (0.88, 1.15)
Intention-to-treat analysis	-	1.05 (0.93, 1.19)
**<u>DR Progression cohort:</u>**		
**Primary analysis**		
60-day grace period, as treated	-2.51 (-9.01, 3.98)	0.92 (0.76, 1.12)
**Sensitivity analysis**		
30-day grace period	-1.13 (-8.51, 6.26)	0.97 (0.79, 1.20)
Intention-to-treat analysis	-	0.93 (0.78, 1.12)

*CI, confidence interval; DR, diabetic retinopathy; GLP1RA, glucagon-like peptide-1 receptor agonists; NPDR, non-proliferative diabetic retinopathy; SGLT2i, sodium-glucose cotransporter-2 inhibitors. *per 1,000 person-years.*
